# Anomaly detection using spatial and temporal information in multivariate time series

**DOI:** 10.1038/s41598-023-31193-8

**Published:** 2023-03-16

**Authors:** Zhiwen Tian, Ming Zhuo, Leyuan Liu, Junyi Chen, Shijie Zhou

**Affiliations:** School of Information and Software Engineering, University of Electronic Science and Technology of China, Chengdu, 610054 China

**Keywords:** Computer science, Software

## Abstract

Real-world industrial systems contain a large number of interconnected sensors that generate a significant amount of time series data during system operation. Performing anomaly detection on these multivariate time series data can timely find faults, prevent malicious attacks, and ensure these systems safe and reliable operation. However, the rarity of abnormal instances leads to a lack of labeled data, so the supervised machine learning methods are not applicable. Furthermore, most current techniques do not take full advantage of the spatial and temporal dependencies implied among multiple variables to detect anomalies. Hence, we propose STADN, a novel Anomaly Detection Network Using Spatial and Temporal Information. STADN models the relationship graph between variables for a graph attention network to capture the spatial dependency between variables and utilizes a long short-term memory network to mine the temporal dependency of time series to fully use the spatial and temporal information of multivariate time series. STADN predicts the future behavior of each sensor by combining the historical behavior of the sensor and its neighbors, then detects and locates anomalies according to the prediction error. Furthermore, we improve the proposed model’s ability to discriminate anomaly and regularity and expand the prediction error gap between normal and abnormal instances by reconstructing the prediction errors. We conduct experiments on two real-world datasets, and the experimental results suggested that STADN achieves state-of-the-art outperformance.

## Introduction

As the rapid growth of communication technology and the continuous enhancement of computing and storage capabilities of embedded devices such as sensors and processors, the application of network communications and embedded devices in real-world systems has increased sharply. Therefore, it is critical to defend against malicious activity in systems and networks and improve the reliability of real-world systems. The absence of appropriate detection and defense measures may lead to tremendous financial losses^1^ and even catastrophic consequences for society. For instance, British Airways IT systems failure affected its ability to check passengers’ boarding, costing 58 million GBP in lost business and follow-up compensation claims^2^. Across England and Wales, nearly 3 billion l of water are lost to spills daily, causing serious waste of resources and huge economic losses^3^.

Real-world industrial systems contain a large number of interconnected sensors that generate a significant amount of time series data during system operation. Performing anomaly detection on these multivariate time series data can timely find faults, prevent malicious attacks, and ensure these systems safe and reliable operation. Anomaly detection, also known as outlier detection or novelty detection, is the process of detecting those data instances that significantly deviate from most data instances^4^. Multivariate time series is a collection of observations for multidimensional variables (or features) recorded in chronological order. Performing unexpected behavior detection on a set of interrelated and interacting observations whose values change over time is called anomaly detection in multivariate time series^5^.

In previous studies, many efforts have been made for anomaly detection, such as nearest-neighbor-based approaches^6–9^, clustering-based approaches^10–13^ and projection-based approaches^14–17^. Nearest-neighbor-based anomaly detection approaches and clustering-based anomaly detection approaches are difficult to scale to large datasets. Projection techniques transform the raw data into a new space with lower dimensionality or complexity, and then perform anomaly detection in the projected space. However, there is no guarantee that the new space can retain appropriate and sufficient information for specific anomaly detection methods. Industrial systems contain production equipment, operating systems, communication networks and various controllers, and are great in scale and complex in composition. The multivariate time series data generated by the sensors in such systems (for brevity, hereafter abbreviated as multivariate time series data) are often characterized by high dimensionality, a long time span, and large data volumes. Therefore, the methods mentioned above are not applicable in this scenario.

Deep learning techniques have powerful data analysis and processing capabilities, especially in complex data (such as high-dimensional data, spatial data, temporal data, and graph data) processing, abstract information mining, and result prediction, making deep learning-based anomaly detection research gradually gaining attention. Deep learning anomaly detection, referred to as deep anomaly detection, aims to perform anomaly detection by learning feature representations or anomaly scores through neural networks^4^. There are numerous studies in the case of deep anomaly detection in recent years. For instance, autoencoders (AE) are the commonly used anomaly detection techniques that directly use the data reconstruction error as anomaly scores^18–21^. Generative adversarial networks (GANs) capture the normality of the given data, and then gets the anomaly scores by defining some form of residuals between the Original and generated instances^22–25^. While these techniques have shown power in locating outliers in tabular data format, they inherently ignore the intrinsic associations between objects.

Anomaly detection in multivariate time series faces severe challenges. (1) Since the rarity of outlier instances, labeled data is generally lacking, which leads to the inability to obtain large-scale labeled data in most scenarios (**challenge 1**). (2) The multivariate time series data connote the complex relationship between sensors, so identifying intricate feature interactions and couplings is a very essential work in multivariate time series anomaly detection (**challenge 2**). (3) Anomalies usually exhibit obvious abnormal characteristics in low-dimensional space, but they become hidden and inconspicuous in high-dimensional space, while industrial systems have numerous devices and the data they generate are usually high-dimensional (**challenge 3**).

Our work, aiming at the above problems and integrating with practical applications, proposed a novel Anomaly Detection Network Using Spatial and Temporal Information, termed STADN, which is based on the method of predictability modeling and uses sensor data’s temporal and spatial information to detect anomalies. Architecturally, STADN has two major components: a Predictive Module (PM), and an Error Refinement Module (ERM). Specifically, in PM, we learn the relationship between sensor pairs, encode them as edges in a graph, and aggregate the behavior of neighbors based on the attention function of adjacent sensors in the graph. Then, we combine the aggregated neighbor behavior and its own historical behavior to predict the future behavior of each sensor. To achieve accurate predictions, ERM reconstructs the prediction error generated by PM, then uses the reconstructed prediction error to refine the rough prediction value of PM to acquire a more accurate prediction value.

Based on our work, the following innovations and contributions can be summarized.

- Proposed STADN. STADN simultaneously captures the temporal and spatial dependencies of multivariate time series data (address challenge 2), uses a predictability modeling-based approach for anomaly detection (address challenge 1 and challenge 3), and helps users locate sensors where anomalies occur, enabling them to quickly diagnose and compensate to anomalies.
- Proposed a prediction error refinement strategy. Improved our model’s ability to discriminate anomaly and regularity by reconstructing the prediction errors. Addressed the challenge that predictability modeling-based anomaly detection methods face under unsupervised learning conditions, that is, the gap between the abnormal scores (determined by the prediction error) between normal and abnormal instances is too small.
- Evaluated STADN on two publicly available anomaly detection datasets. The experimental results indicate that STADN detects anomalies more accurately than baselines. Further evaluations prove our approach’s ability in locating abnormal sensors.

## Related work

Anomaly detection is usually regarded as an unsupervised learning problem as a result of the dearth of labeled outlier instances. Over the past decades, researchers have developed a large number of classical unsupervised methods, among which the predictability modeling-based approaches stand out. The Predictability modeling-based methods use the representations of previous instances within a temporal window as the context to predict the current/future data instances, thus learning the feature representations^4^. These representations capture the temporal/sequential and recurrent dependencies within a given sequence length for accurate predictions. Normal instances typically follow these dependencies closely and can be predicted accurately, while anomalies tend to violate these dependencies and are unpredictable. Hence, the prediction error indicates the degree of the anomaly of a given instance. Methods of this type address the problems of novelty detection in high-dimensional and temporal data by intentionally learning expressive low-dimensional representations with temporal dependency. The predictability modeling-based approaches have two main advantages. First, this method can be integrated with many sequence learning techniques. Second, this method makes it possible to learn different types of temporal and spatial dependencies.

Long short-term memory (LSTM) networks capture temporal dependency, including stationary and non-stationary dynamics as well as short-term and long-term dependencies, and represent them in low-dimensional state representations^26^. LSTM networks perform exceptionally well in modeling time series and time-variant systems^27^. Many LSTM-based anomaly detection methods^28–31^ that have emerged in recent years have proved that LSTM networks have excellent anomaly detection capability. However, most methods ignore learning spatial dependencies, thus facing difficulties in modeling multivariate time series generated by sensors with potential interrelationships. This deficiency limits their ability to detect, locate and interpret anomalies as they occur. To remedy this defect, we introduce graph neural networks to sufficiently learn the intricate correlations between variables in multivariate time series.

Graph neural networks (GNNs) enable deep learning on graph-structured data and play an important role in many research directions. GNNs first identify the nodes and edges of the data, then converts the graph into features for neural networks. In other words, each node in GNNs aggregates the features of its neighbors via the message passing mechanism to compute its own feature representation. Through years of research and development, several different variants of GNNs have been created, including graph convolution networks (GCNs)^32^, graph attention networks (GATs)^33^ and multi-relational approaches^34^. Among them, GATs are applicable to the case where nodes have different weights to their neighbors, that is, when computing the aggregation features of the central node, each neighbor’s contribution is assigned different importance.

## Proposed method

### Problem statement

Given the sensor data (i.e., multivariate time series data) of *N* sensors on *T* time ticks, which is denoted as $S = \left[ S_1, S_2, \ldots , S_T \right]$, $S_t \in \mathbb {R}^N$. In each time tick *t*, $S_t = \left[ s^{(t)}_1; s^{(t)}_2; \ldots ; s^{(t)}_N \right]$ representing the values of *N* sensors. At time tick *t*, our method takes the historical time series data within a sliding window of size *W* as the input $X_t \in \mathbb {R}^{N \times w}$ and outputs the predicted sensor data at the current time tick, i.e., $\hat{S}_t$. Users can detect whether time tick *t* is abnormal according to the prediction error *Err*. If so, one or several abnormal sensors can be located.

1 $$\begin{aligned} X^{(t)} := \left[ S_{(t-w)}, S_{(t-w+1)}, \ldots , S_{(t-1)} \right] \end{aligned}$$

### Overview

**Figure 1:**
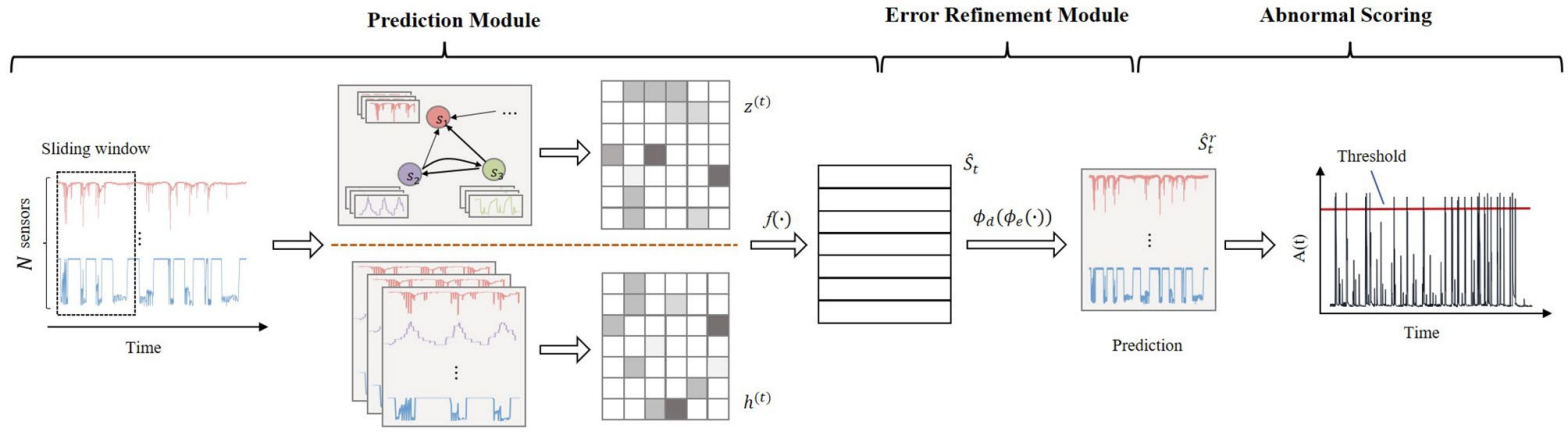
Overview of our proposed framework.

We propose STADN to deal with the challenges faced by anomaly detection in multivariate time series. As illustrated in Fig. 1, STADN contains two essential components: Prediction Module (PM) and Error Refinement Module (ERM). PM generates prediction data $\hat{S}_t$ from previous data within a temporal window. ERM reconstructs the prediction error $E_t$ and adds a refinement $\hat{E}_t$ to $\hat{S}_t$ to obtain reconstructed data $\hat{S}_t^r$. In the following subsections, we will elaborate on these two modules.

### Predictive module

#### Spatial dependency modeling based on GAT

In reality, the behavior of some sensors is intrinsically correlated. We represent the sensor data as a directed graph, represented by the adjacency matrix *A*, with the sensors mapped as nodes in the graph. The directed edges connect the target node to a set of source nodes that are the nearest neighbors of that target node. The edge between the target node and the source node represents sensor dependency relationships. It should be emphasized that neighbors refer to other sensors that have dependency relationships. For a given sensor, we use the information of its nearest neighbors to model the sensor’s behavior.

**Figure 2:**
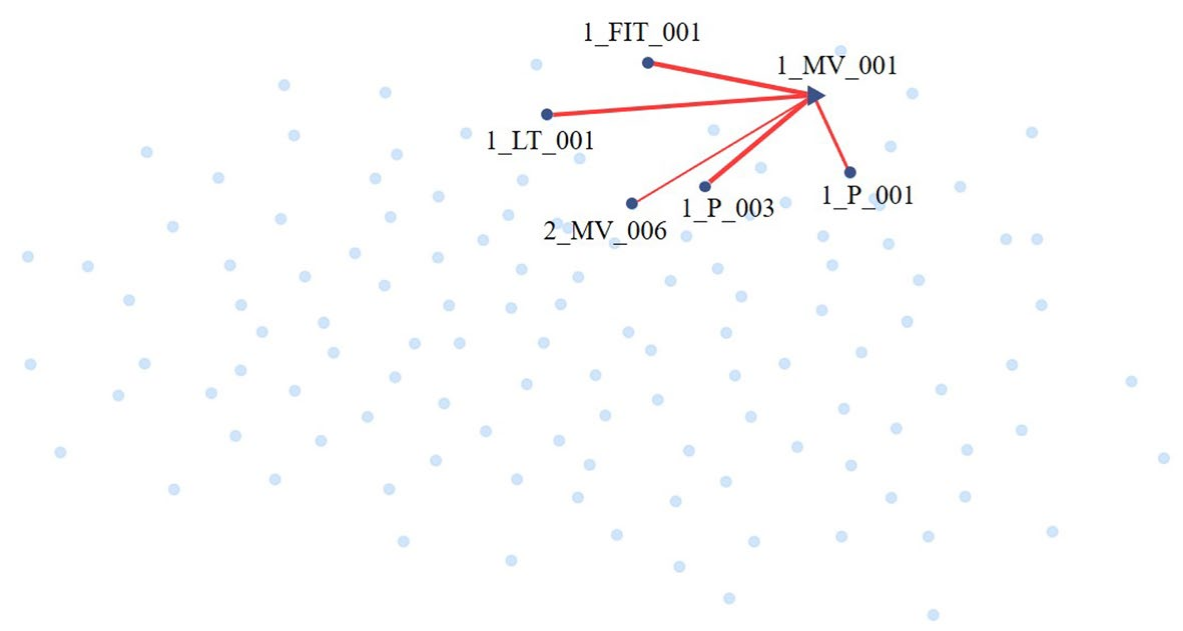
Find out the nearest neighbor set $\mathscr {N}\left( {1\_{\text {MV}}\_001}\right) = \{{1\_{\text {P}}\_003, 1\_{\text {FIT}}\_001, 1\_{\text {LT}}\_001, 1\_{\text {P}}\_001, 2\_{\text {MV}}\_006} \}$ of sensor 1_MV_001 in the WADI dataset by selecting the top 5 $e_{j,i}$.

Define sensor *i* ( denoted as $s_i$) as the target node *i* and node *i* is connected to a source node *j* via an edge (*j*, *i*), where $s_j$ is in the set of *k* nearest neighbors of $s_i$. The edge feature vector represents the degree of dependence between the two sensors:

2 $$\begin{aligned}{} & {} e_{i,j} = d\left( s_i, s_j \right) \end{aligned}$$

3 $$\begin{aligned}{} & {} A_{ji} = 1 \left\{ j \in \text {TopK} \left( \left\{ e_{ki} : k \in \mathscr {V}_i \right\} \right) \right\} \end{aligned}$$

where $0 \le d\left( s_i, s_j \right) \le 1$ represents the distance between $s_i$ and $s_j$, $\mathscr {V}_i=\left\{ 1,2, \cdots , N \right\} - \left\{ i \right\}$ denotes candidate neighbors of $s_i$, all other sensors except $s_i$. If the $s_i$ and the $s_j$ have a dependency relationship, $0 <d\left( s_i,s_j \right) \le 1$, otherwise, $d\left( s_i,s_j \right) = 0$. First, calculate $e_{j,i}$ of $s_i$ on all other sensors, then select the top $\text {K } e_{j,i}$, find the nearest neighbors ${\mathscr {N}}\left( i\right)$ of $S_i$, and construct an adjacency matrix $A_{ji}$. Users can set the value of $\text {K}$ in accordance with the desired sparsity level. As shown in Fig. 2, on the WADI dataset, select the top 5 $e_{j,i}$ and find out the nearest neighbor set $\mathscr {N}\left( 1\_MV\_001\right) = \{{1\_{\text {P}}\_003, 1\_{\text {FIT}}\_001, 1\_{\text {LT}}\_001, 1\_{\text {P}}\_001}, {2\_{\text {MV}}\_006} \}$ of sensor 1_MV_001.

However, in general, we do not have complete prior information about the dependencies of sensor networks. So we propose a flexible method. (1) There is complete prior information about the dependency relationship and dependency degree (that is, we have $d\left( s_i, s_j \right)$, $i, j \in \{1, 2, \ldots , N \}$), then the adjacency matrix is directly constructed according to the prior information. (2) There is no or only some prior information about dependency relationship and degree, then we use Eq. (4) to calculate $d\left( s_i , s_j \right)$.

4 $$\begin{aligned} d\left( s_i, s_j \right) = \left|\rho _{s_i, s_j} \right|+ Similarity \left( s_i, s_j \right) + d\left( s_i, s_j \right) _{prior } \end{aligned}$$

where $\rho _{s_i, s_j}$ is the correlation coefficient between the series data $s_i$ and the series data $s_j$ in a certain multidimensional time series, $Similarity \left( s_i, s_j \right)$ is the behavior similarity between sensors. Here, both $Similarity \left( s_i, s_j \right)$ and $d\left( s_i, s_j \right)$ are normalized. $\left|\rho _{s_i, s_j} \right|$, $Similarity \left( s_i, s_j \right)$, $d\left( s_i, s_j \right) _{prior }$ and $d\left( s_i, s_j \right)$ all take values in the range [0,1].

To fully utilize the spatial structure information of the sensor networks to detect anomalies, a graph attention-based feature extractor is introduced to aggregate the information of selected *k* neighbors of nodes based on the learned adjacency matrix *A*. To do this, first compute the attention coefficients $\alpha _{i,j}$ as follows:

5 $$\begin{aligned}{} & {} \pi (i,j) = LeakyReLU \left( a^T W X_j^{(t)} \right) \end{aligned}$$

6 $$\begin{aligned}{} & {} \alpha _{i,j} = \frac{\exp {\left( \pi (i,j) \right) }}{\sum _{k\in \mathscr {N}(i)} \exp {\left( \pi (i,k) \right) }} \end{aligned}$$

where *a* is the learned coefficient vector of the attention mechanism, and $W \in \mathbb {R}^{d \times w}$ is a trainable weight matrix. $LeakyReLU$ is chosen as the nonlinear activation to calculate the attention coefficients. The softmax function in Eq. (6) is used to normalize the attention coefficients. Then attention is performed on adjacent nodes based on the learned attention coefficients to calculate the aggregated representation $z_i$ of node *i*.

7 $$\begin{aligned} z_i^{(t)} = ReLU \left( {\sum _{j \in \mathscr {N}(i)} \alpha _{i,j} W X_j^{(t)}} \right) \end{aligned}$$

where $X_j^{(t)} \in \mathbb {R}^w$ is the input feature of node *j*, and $\mathscr {N}(i)$ is the neighbor set of node *i* selected from the adjacency matrix *A*.

#### Temporal dependent modeling based on LSTM

Time series data have sequential dependency in the temporal dimension. Sensors and other embedded devices rarely stop running and the data they generate are rich in temporal information. To mine the temporal dependency implied in the data, an LSTM-based feature extractor is introduced to model the short-term and long-term behavior of all sensors.

LSTM networks have been shown to learn sequential dependency more easily. The LSTM cell consists of input gate, forget gate and output gate, which are respectively denoted as *g*, *f* and *o*. These three gates control the path of information transfer. Recurrent weights, input weights and biases in LSTM cells are denoted as *W*, *U*, and *b*. They are all learnable network parameters. The forget gate uses Eq. (8) to find out which information from the previous state needs to be retained for further calculations

8 $$\begin{aligned} f_i^{(t)} = \sigma \left( W_f X_i^{(t)} + U_f h_i^{(t-1)} + b_f \right) \end{aligned}$$

where, $\sigma$ denotes the sigmoid activation function, $X_i^{(t)} \in \mathbb {R}^w$ is node *i*’s input feature, and $h_i^{(t-1)}$ is the output of the LSTM cell at the previous moment. After that, the input gate calculates an intermediate parameter $g_i^{(t)}$ using Eq. (9). $g_i^{(t)}$ and $f_i^{(t)}$ together decide whether to write the candidate state $\tilde{c}_i^{(t)}$ (from Eq. (10) to the memory cell)

9 $$\begin{aligned}{} & {} g_i^{(t)} = \sigma \left( W_g X_i^{(t)} + U_g h_i^{(t-1)} + b_g \right) \end{aligned}$$

10 $$\begin{aligned}{} & {} \tilde{c}_i^{(t)} = tanh \left( W_c X_i^{(t)} + U_c h_i^{(t-1)} + b_c \right) \end{aligned}$$

where, $tanh$ is the tanh activation function. Then, the internal state is updated by Eq. (11) for linear recurrent information transfer. Finally, the output gate controls the quantity of information passed from the internal state $c_i^{(t)}$ to the external state $h_i^{(t)}$ at the current moment, and the final output prediction $h_i^{(t)}$ is obtained from Eq. (13).

11 $$\begin{aligned}{} & {} c_i^{(t)} = f_i^{(t)} \cdot c_i^{(t-1)} + g_i^{(t)} \cdot \tilde{c}_i^{(t)} \end{aligned}$$

12 $$\begin{aligned}{} & {} o_i^{(t)} = \sigma \left( W_o X_i^{(t)} + U_o h_i^{(t-1)} + b_o \right) \end{aligned}$$

13 $$\begin{aligned}{} & {} h_i^{(t)} = o_i^{(t)} \cdot tanh \left( c_i^{(t)} \right) \end{aligned}$$

#### Output layer

From the two feature extractors presented above, we learn the spatial dependency and temporal dependency representations of all nodes within a sliding window, i.e., $\left\{ z_1^{(t)}, z_2^{(t)}, \ldots , z_N^{(t)} \right\}$ and $\left\{ h_1^{(t)}, h_2^{(t)}, \ldots , h_N^{(t)} \right\}$, where $z_i^{(t)} \in \mathbb {R}^G$ and $h_i^{(t)} \in \mathbb {R}^L$. We concatenate $z_i^{(t)}$ and $h_i^{(t)}$ as the input of stacked fully-connected layers with $N\text {-dimensional}$ output to predict the sensor values at time *t*, i.e., $\hat{S}_t$:

14 $$\begin{aligned} \hat{S}_t = f \left( z_1^{(t)} \oplus h_1^{(t)}, z_2^{(t)} \oplus h_2^{(t)}, \ldots , z_N^{(t)} \oplus h_N^{(t)} ; \Theta \right) \end{aligned}$$

where $\Theta$ is the parameters that the Prediction Module needs to optimize. STADN chooses the mean squared error between the predicted output $\hat{S}_t$ and the observations $S_t$ as the empirical risk minimization function.

15 $$\begin{aligned} L_\text {MSE} = \frac{1}{T-W} \sum _{t=w+1}^T {\left\| \hat{S}_t - S_t \right\| }_2^2 \end{aligned}$$

### Error refinement module

It is assumed that normal events can be predicted well, i.e., normal events have smaller prediction errors. Therefore, we can use the difference between the expected and observed behavior of the sensor *i* at time *t* for anomaly prediction. Using Eq. (16), one can calculate the error of $s_i$ at time *t*.

16 $$\begin{aligned} Err_i\left( t\right) = \left|\hat{s}_i^t - s_i^t \right|\end{aligned}$$

Higher $Err_i(t)$ indicates that sensor *i* is more likely to be abnormal at time *t*. Therefore, it is possible to predict whether a sensor is normal or abnormal at time *t* based on its score $Err_i(t)$. One can set a threshold to distinguish between normal and abnormal. Since PM simultaneously captures the temporal dependencies and complex relationships between sensors, its representation ability is further improved, enabling it to predict sensor values at abnormal time points well. As a result, *Err* calculated from prediction errors is too small, and users cannot easily find a suitable threshold to pick out anomalies from the data.

We address this issue by proposing a prediction error refinement strategy. In the refinement stage, the prediction error *Err*(*t*) is reconstructed through the Error Refinement Module (ERM) to obtain an estimate $\hat{E}_t$ of *Err*(*t*), as shown in Eq. (17). Finally, sum $\hat{S}_t$ and $\hat{E}_t$ to obtain an accurate estimate $\hat{S}_t^r$ in Eq. (18)

17 $$\begin{aligned}{} & {} \hat{E}_t = \phi _d \left( \phi _e \left( Err(t); \Theta _e \right) ; \Theta _d \right) \end{aligned}$$

18 $$\begin{aligned}{} & {} \hat{S}_t^r = \hat{S}_t + \hat{E}_t \end{aligned}$$

where $\phi _e$ is an encoder with parameters $\Theta _e$ and $\phi _d$ is a decoder with parameters $\Theta _d$. In order to avoid the model relying heavily on the ERM for refinement, the PM and the ERM are trained separately. First, PM is trained by minimizing $L_\text {MSE}$ in Eq. (15), and then REM is trained and optimized after the parameters of PM are fixed.

**Figure 3:**
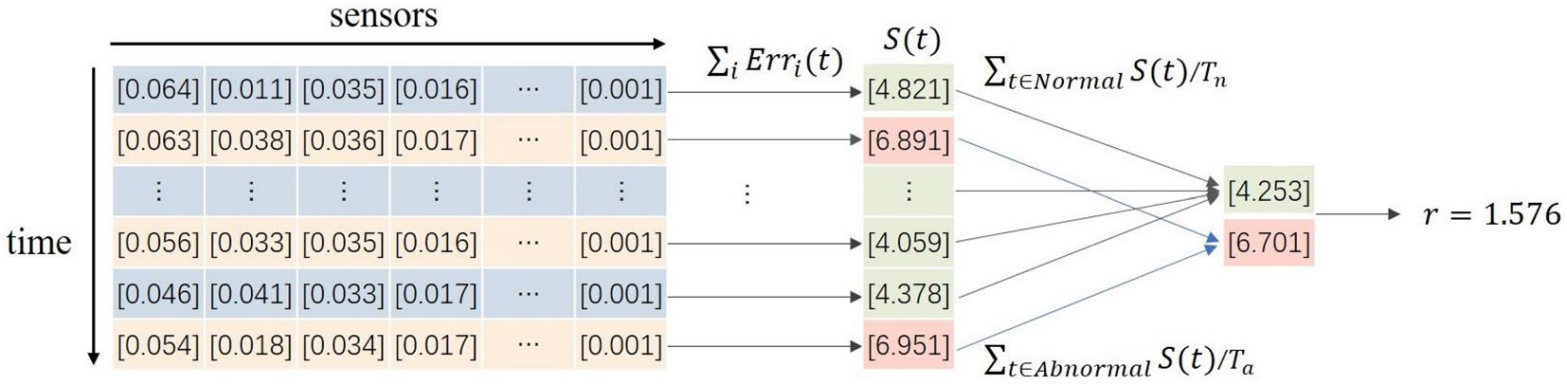
First, *S*(*t*), the sum of *Err* for all sensors at time t, is calculated, and then the ratio of the average of *S*(*t*) for all abnormal times to the average of *S*(*t*) for all normal times is used to quantify the capability of our model. Note that the data in this figure are not experimental results and are only used to illustrate the process of calculating *r*.

We use the regularity score ratio *r* to quantify the model’s ability to discriminate between anomaly and regularity:

19 $$\begin{aligned}{} & {} r =\frac{{\sum _{t \in Abnormal}} S(t) / {T_a}}{{\sum _{t \in Normal}} S(t) / {T_n}} \end{aligned}$$

20 $$\begin{aligned}{} & {} S(t) = \sum _i Err_i(t) \end{aligned}$$

where *S*(*t*) is the regularity score at time tick *t*. *Nnormal* and *Abnormal* are the sequence number sets of normal time ticks and abnormal ones respectively, while $T_n$ and $T_a$ are the total numbers of normal time ticks and abnormal ones separately. To measure the learning ability of our model for each sensor in high-dimensional data, we take the sum of *Err* of all sensors at time *t* as the regularity score in time *t*, as in Eq. (20). $Err_i(t)$ is the difference between the expected and observed behavior of sensor *i* at time *t*. The stronger the model’s capability to discriminate between anomaly and regularity, the larger the difference between $Err_i(t)$ of normal events and $Err_i(t)$ of abnormal events should be. In multivariate time series data, only some sensors are anomalous at certain times. As shown in Fig. 3, *r* can quantify the model’s ability from a global perspective.

### Abnormal scoring

To detect and interpret anomalies in the sensor network, we take the prediction error $Err_i(t)$ in Eq. (16) as the anomaly score for each sensor at each time tick to allow users to locate which sensors are anomalous. Users want to detect anomalous time points. To do this, we combine the individual anomaly scores for each sensor into a single anomaly score for each timescale. Since different sensors may have different characteristics, their prediction errors may also have different scales. We robustly normalize $Err_i(t)$ as Deng^35^ did, thus avoiding any one sensor from producing more bias than the others and minimizing the effect of extreme results

21 $$\begin{aligned} a_i(t) = \frac{\text {Err}_i(t) - \tilde{\mu }_i}{\tilde{\sigma }_i} \end{aligned}$$

where $\tilde{\mu }_i$ and $\tilde{\sigma }_i$ are respectively the median and interquartile range of $Err_i(t)$. The mean and variance of the sample tend to be negatively affected by outliers, while the median and interquartile range are more robust to anomalies and are therefore chosen.

Whenever one sensor has abnormal behavior at time *t*, the time tick *t* will show abnormality. To quantify the anomaly at time *t*, we use the max function to aggregate the sensors’ $a_i (t)$, as in Eq. (22). Therefore, we can evaluate the abnormality of time tick t according to its score *A*(*t*). A threshold is set to distinguish between normal and abnormal, and if *A*(*t*) exceeds it, then the time tick *t* will be marked as an anomaly.

22 $$\begin{aligned} A(t) = \max _i\text { }a_i(t) \end{aligned}$$

## Experiments

To testify the effectiveness of STADN, we perform a series of experiments on two publicly available anomaly detection datasets and compare our method with many existing approaches. We also provide further evaluation and analysis of STADN.

### Datasets

**Figure 4:**
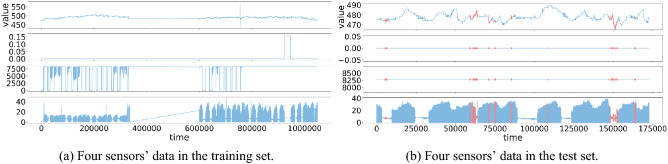
The WADI dataset visual examples. Mark anomalies in red. Note that only four sensors’ data are plotted for the WADI dataset as visual examples.

Here, we briefly describe the two datasets that will be used in the later experiments. The Water Distribution (WADI) dataset, published by the Centre for Research in Cyber Security of Singapore University of Technology and Design, is a distribution system consisting of a more significant number of water distribution pipelines^36^. This dataset collects all data generated by 127 devices operating continuously for 16 days (2 days in an attack scenario with 15 attack types and the remaining days in normal operation). In the training data, WADI contains only normal data. WADI has high dimensionality, and the data is unbalanced. The data of some sensors in WADI is shown in Fig. 4. The Secure Water Treatment (SWaT) dataset was derived from an operational water treatment testbed that is a scaled-down version of a large modern water treatment plant in a major city^37^. The testbed covers an area of about 90 square meters and produces 5 US gallons per hour of filtered water. The data collected in both the WADI and SWaT datasets are composed of two parts, one is collected in normal operation and the other is collected in staged attack scenarios. We use the first part of the data for training and verification and the second part for testing. Detailed datasets statistics are shown in Table 1.

**Table 1:** Datasets statistics.

Datasets	Features	Train	Test	Anomalies (%)
WADI	127	789,371	172,801	5.77
SWaT	51	495,000	449,919	12.14

### Evaluation metrics

To determine anomalies, we first need to determine a threshold. There are generally two ways to determine the threshold. (1) Enumerate the test set and find an optimal global threshold to achieve the maximum F1-score (F1 for short). (2) Set the maximum value of *A*(*t*) on the validation data as the threshold, which is always available for anomaly detection in the absence of significant changes in the data distribution.

Given a specific threshold, we can Compute the True Positives (TP), False Positives (FP), True Negatives (TN), and False Negatives (FN). Thus we have $\text {precision} = \frac{\text {TP}}{\text {TP} + \text {FP}}$, $\text {recall} = \frac{\text {TP}}{\text {TP} + \text {FN}}$, and $\text {F1}= \frac{2 \times \text {precision} \times \text {recall}}{\text {precision} + \text {recall}}$. The Receiver Operating Characteristic (ROC) curve intuitively reflects the trend of sensitivity and specificity of the model when different thresholds are selected. It is not influenced by the choice of the best threshold. Given all possible thresholds, $\text {TPR} = \text {recall} = \frac{\text {TP}}{\text {TP} + \text {FN}}$, $\text {FPR} = \frac{\text {FP}}{\text {FP} + \text {TN}}$, then we have the ROC curve with FPR as the x-axis and TPR as the y-axis.

### Baselines

We compare STADN with seven other anomaly detection methods in terms of performance. These seven methods are either classical or state-of-the-art.

- KNN: Outliers are defined based on distances, i.e., considering the sum of the distances of each point to its *k* nearest neighbors, and those points with the largest values are outliers^6^.
- FB: Feature Bagging approach combines the outlier scores calculated by the individual outlier detection algorithms, each of which uses a small number of randomly selected features from the original feature set^38^.
- AE: Autoencoders consist of an encoding network and a decoding network^18^. It identifies abnormal data based on reconstruction errors.
- DAGMM: Deep Autoencoding Gaussian Mixture Model learns low-dimensional representations of the samples through a compression network, and then evaluates sample energy through an estimation network under the framework of Gaussian Mixture Modeling^20^.
- LSTM-VAE: Long short-term memory-based variational autoencoder projects the inputs and their temporal dependency as latent space representations, thus estimating inputs expected distribution and detecting anomalies by determining whether their log-likelihood is below a threshold^39^.
- MAD-GAN: Multivariate Anomaly Detection with GAN constructs long-short-term-memory recurrent neural network generator and discriminator in the GAN framework, and detects anomalies via discrimination and reconstruction^25^.
- GDN: Graph Deviation Network integrates a structural learning method with graph neural networks for forecasting, and then identifies and explains anomalies by prediction errors^35^.

### Experimental settings

In our experiments, we select ReLU as the nonlinear activation function. All parameters are initialized with Glorot initialization in Predictive Module. In the training process, the Adam optimizer is employed to minimize the loss function with a learning rate of 0.001. We use *k* with 40 (15), *w* with 20 (20) and hidden layers of 256 (128) neurons for the WADI (SWaT) dataset. In order to make the models converge faster, we apply the batch normalization. When the validation accuracy does not increase in 15 consecutive epochs, we stop training early to select the best model.

### Anomaly detection performance and analysis

**Figure 5:**
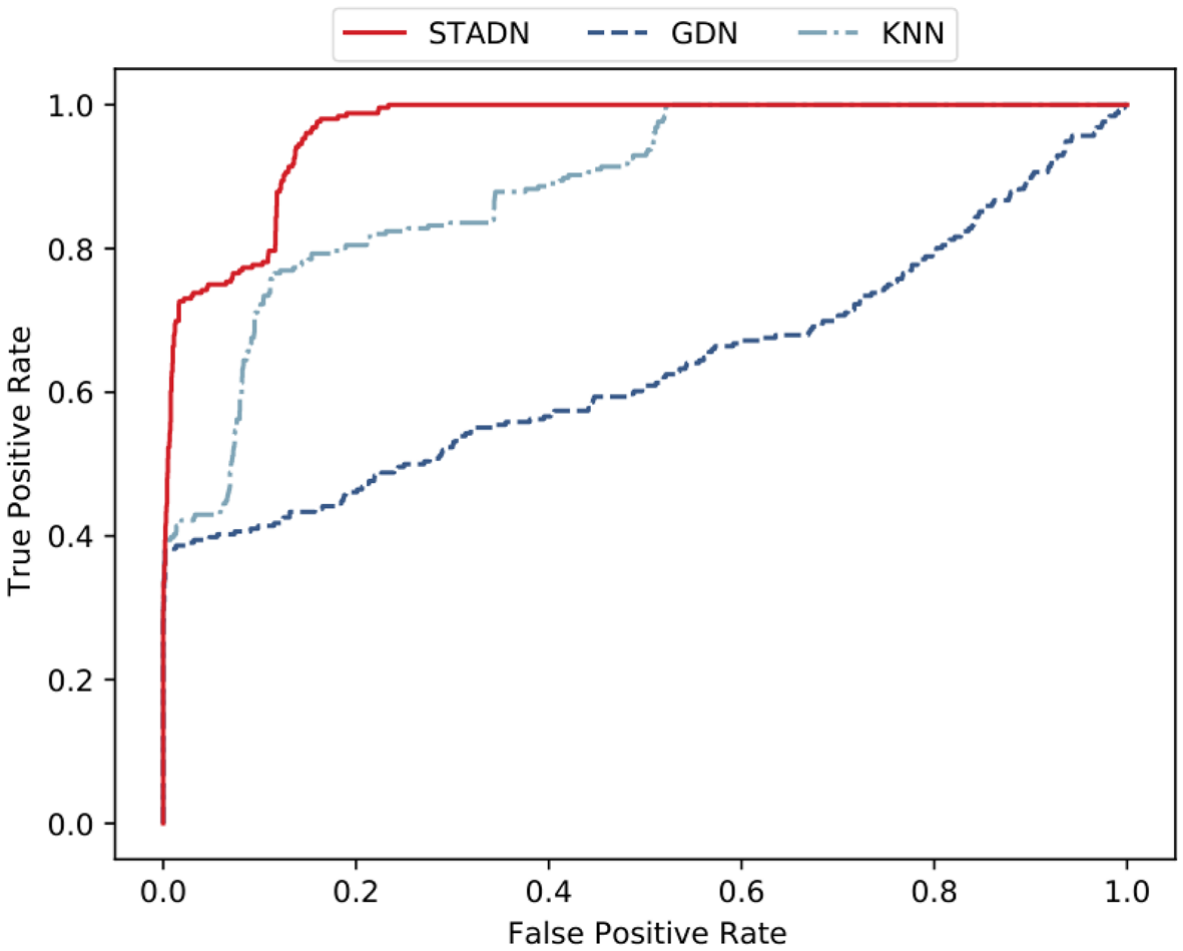
Comparison of the ROC curves of STADN, KNN and GDN on the WADI dataset.

We set the maximum value of *A*(*t*) on the validation data as the threshold to compare the precision, recall and F1 of baselines with STADN, and the results are presented in Table 2. The experimental data show that STADN outperforms all baselines on both datasets, showing significant improvements. The best precision of STADN on the WADI (SWaT) dataset is up to 98.49% (99.92%). In terms of F1, it tops out at 0.62 (0.83), which is 8.77% (2.47%) higher than the optimal method in the baselines. STADN improves recall to 45.57% (70.79%) while achieving high precision. These fully prove the effectiveness of STADN on the anomaly detection task in multivariate time series. Models considering the temporal dependency of time series data (e.g., LSTM-VAE and MAD-GAN) perform better than other models that do not (e.g., KNN and AE). Therefore, considering the temporal dependency of time series data helps to provide better prediction accuracy. For the model that only considers the temporal dependency of high-dimensional time series data, GNN-based models (e.g., GDN and STADN) perform better than other methods (e.g., LSTM-VAE and MAD-GAN). This demonstrates the capability of GNNs in modeling the dependence relationships between sensors. Our model simultaneously captures the temporal dependency of multivariate time series data and complex relationships between sensors, and achieves the best performance. In all baseline approaches, GDN uses the same sliding window input historical data for prediction as our method. However, GDN mainly models the spatial dependency in the data and has a weaker modeling ability for temporal dependency, while our method focuses on spatial dependency as well as temporal dependency. We compare the ROC curves of STADN, KNN(the worst of all baselines) and GDN(the best of all baselines) on the WADI dataset, and as is evident in Fig. 5, STADN significantly outperforms KNN and also slightly outperforms GDN.

**Table 2:** Precision (%), recall (%) and F1-score on two datasets. We show the best performance of all baselines in underlined and the best performance of our method in bold.

Method	WADI			SWaT		
	Prec	Rec	F1	Prec	Rec	F1
KNN	7.76	7.75	0.08	7.83	7.83	0.08
FB	8.60	8.60	0.09	10.17	10.17	0.10
AE	34.35	34.35	0.34	72.63	52.63	0.61
DAGMM	54.44	26.99	0.36	27.46	69.52	0.39
LSTM-VAE	87.79	14.45	0.25	96.24	59.91	0.74
MAD-GAN	41.44	33.92	0.37	98.97	63.74	0.77
GDN	97.50	40.19	0.57	99.35	68.12	0.81
STADN	**98.49**	**45.57**	**0.62**	**99.92**	**70.79**	**0.83**

### Localizing anomalies

We use the maximum $a_i(t)$, the maximum prediction error among all sensors at time *t*, as the anomaly score of time *t*, so our method can localize the sensor where the anomaly occurred. $P_i$ is the probability that the prediction error of the sensor *i* is the anomaly score at time tick *t* in a certain time period $\{u, u+1, \ldots , u+k \}$, that is, $P_i = \frac{ \left|\{t | A(t) = a_i(t)\} \right|}{ \left|\{u, u+1, \ldots , u+k \} \right|}$, $\left|\{ \cdot \} \right|$ indicates the number of elements in set $\{ \cdot \}$. We calculate the $P_i$ of a certain sensor *i* in each abnormal period and the sum of *P* of top 10 nearest neighbors of sensor *i*, i.e., $\sum _{}^{}{P_j}, j \in \text {Top10} \left( \left\{ e_{ki} : k \in \mathscr {V}_i \right\} \right)$. Table 3 shows $P_i$ and $\sum _{}^{}{P_j}$ of each abnormal period on the WADI dataset. Note that we do not know the specific sensor (or sensor set) that causes the anomaly at each abnormal time point. We only give the $P_i$ and $\sum _{}^{}{P_j}$ of those sensors mentioned in the attack description.

**Table 3:** $P_i$(%) and $\sum _{}^{}{P_j}, j \in \text {Top10} \left( \left\{ e_{ki} : k \in \mathscr {V}_i \right\} \right)$(%) of each abnormal period on the WADI dataset. Attack identifier and attack description are from^36^. Note that we do not know the specific sensor (or sensor set) that causes the anomaly at each abnormal time point. We only give the $P_i$ and $\sum _{}^{}{P_j}$ of those sensors mentioned in the attack description.

Attack identifier	Attack description	Sensor *i*	$P_i$	$\sum _{}^{}{P_j}$
1	Motorized valve 1_MV_001 is maliciously turned on, this causes an overflow on the primary tank should reflect on 1_LT_001 and 1_FIT_001.	1_MV_001 1_LT_001 1_FIT_001	0.000 0.232 0.364	0.642 0.066 0.046
2	Flow Indication Transmitter 1_FIT_001 is turned off, and a false reading is seen by PLC for 1_FIT_001. This will turn the chemical dosing pump on while leaving the water level in the primary tank constant. Consequently, the attacker is increasing the level of chemicals inside the water.	1_FIT_001	0.383	0.2
3-4	Stealthy attack. The attacker aims to drain elevated reservoir 2_LT_002. This is done by controlling and manipulating tank level draining and filling speed. 1_AIT_001 Moreover the attacker changes the reading seen by the water quality sensor, which causes the raw water tank to drain.	2_LT_002 1_AIT_001	0.000 0.154	0.051 0.000
5	Turn off valves to consumers 2_MCV_101, 2_MCV_201, 2_MCV_301, 2_MCV_401, 2_MCV_501, 2_MCV_601 consumers will receive no more water.	2_MCV_101 2_MCV_201 2_MCV_301 2_MCV_401 2_MCV_501 2_MCV_601	0.000 0.000 0.000 0.000 0.000 0.000	0.000 0.047 0.012 0.070 0.047 0.000
6	Turn on maliciously 2_MCV_101, 2_MCV_201.	2_MCV_101 2_MCV_201	0.000 0.000	0.000 0.000
7	Supply contaminated water to the Elevated Reservoir tank by setting 1_AIT_002 to 6 to drain the primary grid because of contamination. at the same time open 2_MV_003.	1_AIT_002 2_MV_003	0.472 0.000	0.000 0.029
8	Maliciously open 2_MCV_007 in order to produce water leakage before the water reaches consumers. This attack should be reflected in 2_PIT_002 and 2_FIT_002.	2_MCV_007 2_PIT_002 2_FIT_002	0.915 0.000 0.000	0.071 0.017 0.000
9	Turn on 1_P_006 maliciously to cause a pipe burst.	1_P_006	0.800	0.000
10	Damage 1_MV_001 and raw water pump to drain Elevated Reservoir tank.	1_MV_001	0.073	0.585
11	Similar to attack 8.	2_MCV_007 2_PIT_002 2_FT_002	0.927 0.000 0.000	0.044 0.015 0.000
12	Similar to attack 8.	2_MCV_007 2_PIT_00 2_FT_002	1.000 0.000 0.000	0.000 0.000 0.000
13	Reducing Booster set point pressure causes intermittent water supply to consumers this should be reflected in 2_FIT_003 and 2_PIT_003.	2_FIT_003 2_PIT_003	0.000 0.000	0.000 0.000
14	Stop chemical dosing to the raw water which is supplied to the primary grid tank.	–	–	–
15	Stealthy attack, the inverse of attack 3.	2_LT_002 1_AIT_001	0.016 0.000	0.036 0.062

During attack 1, the 1_MV_001 was maliciously turned on, resulting in an anomaly. The attack description of the dataset said that the abnormality would be directly reflected on 1_LT_001 and 1_FIT_001. The probability of STADN locating to the nearest neighbor of 1_MV_001 is 64.2%, and the probability of STADN successfully locating to 1_LT_001 and 1_FIT_001 is 23.2% and 36.4%, respectively. As shown in Fig. 2, on the WADI dataset, select the top 5 $e_{j,i}$ and find out the nearest neighbor set $\mathscr {N}\left( 1\_MV\_001\right) = \{\text {1\_P\_003, 1\_FIT\_001, 1\_LT\_001, 1\_P\_001,} \text {2\_MV\_006} \}$ of sensor 1_MV_001. STADN can successfully find the sensors most closely related to the 1_MV_001 sensor. During attack 9, 1_P_006 was maliciously turned on, and the probability of STADN successfully locating 1_P_006 is as high as 80%. During attack 12, 2_MCV_007 was maliciously turned on, and the probability of STADN successfully locating 2_MCV_007 is as high as 100%. STADN has been proven to not only detect anomalies, but also help users locate the sensor where the anomaly occurs, allowing them to quickly diagnose and compensate for anomalies. STADN detects and locates anomalies by modeling the graph structure between sensors, aggregating neighbor information, and capturing the impact of abnormal sensors on its neighbors.

### Ablation studies

We performed ablation studies using several different STADN variants to further verify the effectiveness of those designs described in “Proposed method”. We removed the Spatial Dependency Modeling based on GAT (denoted as SDM), the Temporal Dependent Modeling based on LSTM (denoted as TDM) and the Error Refinement Module respectively to observe changes in model performance. We name these three variants as *w/o* SDM, *w/o* TDM, and *w/o* ERM (i.e., PM only). The results are given in Table 4.

**Table 4:** Precision (%), recall (%), F1-score and regularity score ratio *r* of STADN and its variants on two datasets.

Method	WADI				SWaT			
	Prec	Rec	F1	*r*	Prec	Rec	F1	*r*
STADN	98.49	45.57	0.62	66.12	99.92	70.79	0.83	33.15
w/o ERM	93.46	40.23	0.55	37.29	94.05	67.38	0.79	15.62
w/o SDM	85.48	41.41	0.56	26.76	96.18	62.71	0.77	18.13
w/o TDM	87.41	43.36	0.58	29.01	95.89	61.83	0.75	16.31

**Figure 6:**
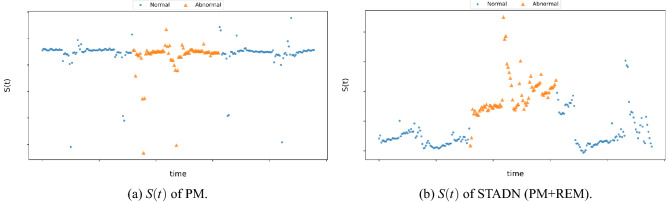
Comparison the *S*(*t*) of PM (w/o ERM) and STADN in the same period on the WADI dataset: the *S*(*t*) of PM at the abnormal time is not larger than most normal times, and users cannot set an appropriate threshold; while the *S*(*t*) of STADN at the abnormal time is larger than most normal times, one can easily find a valid threshold.

The experimental results show that when we remove any part, the performance on two datasets will be degraded. WADI inherently has a higher dimensionality than SWaT and is also more unbalanced than the SWaT dataset, as is evident in Table 1. The variant w/o SDM and the variant w/o TDM have the largest drop in terms of precision on the WADI dataset, suggesting that modeling the temporal and topological dependencies of the data simultaneously helps learn richer representations that capture the temporal/sequential and recurrent dependencies of a sensor as well as spatial dependencies between different sensors. Normal instances typically follow these dependencies closely and can be predicted accurately, while anomalies tend to violate these dependencies and are unpredictable. The variant w/o ERM does not perform as well as the original model and its F1 and recall values are lower. This means that reconstructing the prediction error can refine the model to learn a better representation of normal data, resulting in lower *Err* for normal instances and higher *Err* for abnormal instances (in this case, the value of *r* is the largest), and improve the model’s capability to discriminate between anomaly and regularity. We know a continuous period of abnormal time, and the *S*(*t*) of the original model STADN and the variant *w/o* ERM in this time period are presented in Fig. 6. In Fig. 6a, the *S*(*t*) at the abnormal time is not larger than the *S*(*t*) of most normal times, and users cannot set an appropriate threshold; whereas in Fig. 6b, the *S*(*t*) at the abnormal time is larger than the *S*(*t*) of most normal times, users can easily find a valid threshold.

## Conclusion

We propose STADN, which simultaneously captures the temporal dependency of multivariate time series data and complex relationships between sensors, using a predictability modeling-based method to obtain anomaly scores for instances. STADN solves the problem of too small gaps in anomaly scores (determined by prediction error) between normal and anomalous instances when using the prediction model for anomaly detection by reconstructing the prediction error. The experiments conducted on two publicly available datasets suggest that STADN outperforms all baselines and achieves superior results. STADN can help users detect and locate abnormal sensors, enabling them to quickly diagnose and compensate for anomalies. In future work, we may consider using other sequence learning techniques and dynamic anomaly threshold methods to further improve the applicability of anomaly detection.

## Data Availability

The datasets analyzed during the current study are available at https://itrust.sutd.edu.sg/itrust-labs_datasets/.
